# The role of satellite DNAs in repeatome organization and sex chromosome evolution in the owl monkey *Aotus infulatus* and congeneric species

**DOI:** 10.1186/s13062-026-00948-4

**Published:** 2026-08-27

**Authors:** Gustavo Akira Toma, Edivaldo Herculano Corrêa de Oliveira, Klebson Demelas Maurício, Liliane Almeida Carneiro, Thomas Liehr, Marcelo de Bello Cioffi

**Affiliations:** 1 Department of Genetics and Evolution, Federal University of Sao Carlos, Sao Carlos, SP 13565-905 Brazil; 2 Laboratory of Cytogenomics and Environmental Mutagenesis, Seção de Meio Ambiente, Evandro Chagas Institute, Ananindeua, PA 67030-000 Brazil; 3 Faculty of Natural Sciences, ICEN, Federal University of Pará, Belém, PA 66075-110 Brazil; 4 National Primate Center, Ananindeua, PA 67030-000 Brazil; 5 Institute of Human genetics, Jena University Hospital, 07747 Jena, Germany

**Keywords:** Multiple sex chromosome, Repetitive DNA, Neotropical monkeys

## Abstract

**Background:**

Multiple sex chromosome systems provide exceptional models for investigating the genomic mechanisms underlying sex chromosome evolution. In Neotropical primates, owl monkeys (*Aotus*) are notable for their extensive karyotypic diversity and recurrent emergence of complex sex chromosome systems. Here, we present a comprehensive cytogenomic analysis of the repeatome of *Aotus infulatus*, a species exhibiting an X_1_ × _2_Y sex chromosome system. Using low-coverage whole-genome sequencing from males and females, combined with comparative analyses of three congeneric species (*A. nancymaae*, *A. vociferans*, and *A. trivirgatus*), we characterized the composition, abundance, and evolutionary dynamics of their repeatomes across the genus.

**Results:**

Repetitive sequences account for approximately 37% of the *A. infulatus* genome, with satellite DNAs (satDNAs) representing the most variable component among species. Nine satDNA families were identified in *A. infulatus*, most of which show high sequence conservation across *Aotus*, consistent with a shared ancestral repeat library followed by lineage-specific amplification. Comparative analyses between sexes revealed a modest enrichment of satDNAs in the male genome. Fluorescence in situ hybridization uncovered differential chromosomal distributions of several satellites, including sex-biased accumulation on the X_1_ and Y chromosomes. In contrast, comparative genomic hybridization revealed extensive sharing of repetitive sequences between sexes, indicating limited genomic divergence between the X_1_ and Y chromosomes.

**Conclusions:**

Our results demonstrate that repetitive DNA, particularly satDNAs, has played a central role in reshaping the sex chromosome architecture of *A. infulatus*. This study establishes *Aotus* as a compelling primate model for investigating the genomic consequences of sex chromosome fusion and highlights the importance of repeatome dynamics in the evolution of multiple sex chromosome systems.

**Supplementary Information:**

The online version contains supplementary material available at 10.1186/s13062-026-00948-4.

## Background

 Sex chromosomes are genomic elements that differ between the sexes, often harboring sex-linked or sex-determining genes [1–3]. Their origin is tightly linked to autosome evolution, beginning when an autosome acquires a sex-determining locus [2]. The subsequent accumulation of sexually antagonistic sequences drives the differentiation of homologous chromosomes into distinct X and Y, in the case of male heterogamety, or Z and W chromosomes in female heterogamety. This divergence, together with the progressive reduction of recombination between homologs, facilitates the amplification of repetitive DNAs, such as satellite DNAs (satDNAs), and heterochromatin, ultimately leading to chromosomal degeneration and potential loss of genetic content of sex-limited chromosome [2, 4, 5].

Multiple sex chromosomes are often generated through fusions/translocations between an ancestral sex chromosome and an autosome [6–8]. As a result, species with multiple sex chromosome systems differ in diploid number between males and females and this process produces a neo-sex chromosome, which may subsequently continue to follow the canonical steps of sex chromosome evolution [6, 8]. However, by effectively reorganizing existing linkage groups, the addition of novel genomic blocks via chromosome translocation alters the nucleotide content of sex chromosomes, which may in turn reshape their differentiation trajectory [3, 7–9].

In viviparous mammals, males are the heterogametic sex, characterized by a shared XY/XX sex chromosome system [10, 11]. The Y chromosome carries the *Sry*, a *Sox-3* derived sex determining gene, which acts as a key regulator in the development of the testis [10, 11]. Multiple sex chromosomes have been reported in approximately 152 extant therian species [12]. Notably, Neotropical primates (Platyrhini) account for about 9% of these reports, with occurrences documented in families such as Aotidae, Atelidae, and Callitrichidae [11–13]. Among these, Aotidae stands out as a particularly valuable model for studying sex chromosome evolution. These monkeys, commonly known as Owl monkeys, exhibit a high rate of chromosome rearrangements as demonstrated by their wide diploid number (2n) variation, ranging from 46 to 58 chromosomes [13–15]. Additionally, the existence of congeneric species with both conventional and multiple sex chromosome systems provide a unique opportunity to identify the translocated chromosomes involved in the newly formed sex chromosomes and potentially investigate their evolution [13, 14].

Owl monkeys are a monophyletic group composed of approximately 13 valid species, grouped in only one genus (*Aotus*) [16, 17]. Their interspecific phylogenetic tree is still unresolved, with the existence of at least two distinct species complexes (i) the *azarae* group, with *Aotus azarae*; *A. boliviensis* and *A. infulatus* and (ii) the *lemurinus* group composed of *A. griseimembra*, *A. lemurinus* and *A. zonalis* [16, 17]. From these 13 species, three exhibit an X₁X₂Y sex chromosome system, specifically in the Neotropical owl monkeys (family Aotidae), *A. boliviensis* (2n = 49♂/50♀), *A. infulatus* (2n = 49♂/50♀), and *A. nigriceps* (2n = 51♂/52♀) [12, 14, 17]. Extensive investigations have been conducted on the chromosome evolution and sex chromosomes of *Aotus* [14, 15, 18–20]. However, only a handful delved into the origin and evolution of multiple sex chromosomes in the owl monkeys. Notably, by localizing whole chromosome probes from humans, Araujo et al. [14] identified numerous chromosome rearrangements in *A. infulatus*, among which two were found to be exclusive to this species. This study also proposes that the Neo-Y chromosome found in *A. infulatus* likely arose from a fusion of ancestral chromosomes 16 and Y, producing a conspicuous heterochromatic cluster of three distinguishable bands [14]. Nevertheless, the repetitive DNA composition of these heterochromatic regions and their role in the evolution of *A. infulatus’* multiple sex chromosomes remain largely unexplored.

Due to the common accumulation of repetitive DNAs on sex chromosomes, characterization of the repeatome, a comprehensive catalog of repetitive DNA in a genome, offers a unique opportunity to study sex chromosomes [21–24]. These DNA sequences encompass a huge diversity of categories, ranging from tandem array repeats (e.g., satDNA, multigene families) to interspersed sequences, such as transposable elements (TEs), representing highly informative chromosomal markers [25, 26]. Due to their direct involvement in the structural and functional reshaping of sex chromosomes, repetitive DNA provides crucial insights into the genomic framework underlying sex chromosome differentiation, including the formation of neo-sex chromosomes [2, 9, 27, 28]. Repeatome analysis focusing on multiple sex chromosomes has been conducted in several different organisms [23, 24, 29, 30], allowing the characterization of sex chromosomes’ repeat landscapes.

Here, we investigate the role of repetitive DNAs in the origin and differentiation of the X₁X₂Y sex chromosome system of *Aotus infulatus*. We hypothesize that (i) satDNAs have undergone sex-biased amplification associated with the emergence of neo-sex chromosomes, and (ii) their chromosomal redistribution has contributed to the structural reorganization of the X₁ and Y chromosomes following autosome–sex chromosome fusion. To test these hypotheses, we employed a cytogenomic approach combining repeatome profiling of males and females with in situ mapping of satDNAs and comparative genomic hybridizations (CGH). In addition, we performed comparative analyses with three congeneric species for which suitable genomic sequencing data are currently available (*A. nancymaae*, *A. vociferans*, and *A. trivirgatus*) to place these patterns in an evolutionary context. Together, this integrative framework allows us to evaluate how repeatome dynamics have shaped the evolution of multiple sex chromosomes in *Aotus*.

## Methods

### Sampling and chromosome obtainment

We sampled 16 individuals (seven females and nine males) of *A. infulatus* from the Centro Nacional de Primatas (National Primate Center, Ananindeua - Pará, Brazil; 01°23’04.4” S, 48°22’59.2” W). These individuals were born at the center, are unrelated, and descended from animals originating from the Tucurui region (Belém - Pará, Brazil). Specimen sex was identified based on morphological criteria. To obtain mitotic chromosomes, we performed lymphocyte cell cultures following De Oliveira et al. [31]. Heparinized peripheral blood was taken from each individual and added to the culture medium (RPMI-1640) supplemented with phytohemagglutinin, 15% fetal bovine serum, L-glutamine, penicillin, and streptomycin. Cells were maintained at 37 °C, and after 72 h, colchicine (0.0125%) was added to the cultures for 30 min. The cells were then harvested, treated with KCL (0.075 M), and fixed with Carnoy I fixative (3:1 methanol/glacial acetic acid). Chromosome slides were stained with an 8% Giemsa solution (pH 6.8) for karyotype analysis. All experiments were conducted in strict accordance with the ethical guidelines and regulations of the Animal Experimentation Ethics Committee of the Evandro Chagas Institute (CEUA-IEC 24/2022). Importantly, no animals were euthanized for this study; all procedures relied exclusively on minimally invasive blood sampling from living individuals.

## DNA extraction and short-reads sequencing data

Peripheral blood samples from one female and one male of *A. infulatus* were collected in vacuum tubes containing EDTA, and genomic DNA was extracted using the Wizard^®^ Genomic DNA Purification Kit (Promega), following the manufacturer’s instructions. Paired-end libraries of 150pb read length were constructed from each gDNA, and low coverage sequencing was performed on the BGISEQ-500 platform at BGI (BGI Shenzhen Corporation, Shenzhen, China). The sequencing yielded a total of 1.2Gb of raw reads from males and females. All genomic reads were deposited in the sequencing read archive (SRA) under accession numbers SRR36746884 (male) and SRR36746885 (female).

In addition, publicly available sequencing datasets from three congeneric species (*A. nancymaae* [SRR7608132], *A. vociferans* [ERR10943613], and *A. trivirgatus* [ERR10941484]) were incorporated into the repeatome analysis from the NCBI-SRA. Prior sex information was available only for *A. nancymaae*, which corresponded to a male individual. To determine the sex of the other two *Aotus* species, we performed a Blastn search using the mRNA of three Y-specific genes (SRY, XM_035289950.1; RMBY, NM_001267757.1; TSPY, XM_078364512.1) retrieved from *Callithrix jacchus*. Detectable hits were recovered only for *A. vociferans*, consistent with a male origin, whereas no hits were observed for *A. trivirgatus*, supporting its classification as female.

### Characterization of *Aotus* repeatome

To characterize the whole satDNA libraries (satellitome) of the four *Aotus* species (*A. infulatus*, *A. nancymaae*, *A. vociferans*, *A. trivirgatus*), we used the Tandem Repeat Analyzer (TAREAN) tool within the RepeatExplorer2 [32], which identifies satDNA repeats based on the topology of cluster graphs, in combination with the satMiner protocol [33]. First, we improved the quality of sequencing reads in trimmomatic version 3.0 [34], and by using the parameters: LEADING:3, TRAILING:3, SLIDINGWINDOW:4:20, MINLEN: 100 CROP, we removed sequencing adapters and low-quality reads/nucleotides (Q < 20). Then, to normalize satDNA recovery across libraries a random subset of 2 × 500,000 paired reads from the male genome of *A. infulatus* and the three remaining species were produced and individually loaded into TAREAN. TAREAN analysis was performed using default parameters, with minimum cluster size set to 0.001% and running queue specified as extra-long. We selected putative satDNAs with similar circular topology graphs and used the consensus monomer sequence generated by TAREAN for downstream analysis. In the next step, satDNAs recovered were filtered from their respective trimmed reads by using Deconseq version 0.4.3 [35]. A new subsample of 2 × 500,000 paired reads was performed for each sequencing library and used in multiple rounds of TAREAN and Deconseq filtering until no novel satDNA were found. Finally, we carried out a screening of non-satDNA repetitive DNA (e.g., multigene families, TEs) from each satellitome dataset. All catalogs of satDNAs were deposited on the Genbank with accession numbers: PX852210-PX852218 (*A. infulatus*), PX852219-PX852226 (*A. nancymaae*), PX852236-PX852244 (*A. vociferans*) and PX852227-PX852235 (*A. trivirgatus*).

The relative proportions of different repetitive DNA categories, as well as the annotation of TEs, were determined using the DNApipeTE software (version 1.4) [36]. This pipeline enables comprehensive repeat surveys from low-coverage sequencing data without requiring a reference genome assembly. As input, we selected single-end forward reads of each trimmed sequencing library and launched DNApipeTE with default parameters using a genome coverage of 0.1x. To estimate the genome proportion of each repetitive DNA class, we used the reference genome size of 2.7Gb, based on previous genome assemblies of closely related species (GCA_963574785.1; GCA_963574505.1; GCA_963573465.1; GCA_963573775.1; GCA_000952055.2; GCA_048565385.1). However, considering that assembly lengths can be affected by systematic artifacts such as collapse of repetitive regions or unresolved duplications/haplotigs, in the absence of independent genome size validation, we opted for a conservative approach using equal-sized read samples (2 × 5,000,000 paired-end reads) rather than normalizing by assembled genome lengths, which could otherwise introduce comparative bias across species. Graphical representations were generated using the R package ggplot2 [37] and combined with the *Aotus* phylogeny using ggtree [38].

### Estimating the abundance, divergence, and homology of satDNAs

The abundance of each satDNA (number of nucleotides related to the particular satDNA vs. total number of analyzed nucleotides in the determined genome) was calculated by using normalized random 2 × 5,000,000 paired reads from each species’ genome, including males and females of *A. infulatus*. We used the “cross-match” tool in RepeatMasker [39] version 4.1.6 and a custom Python script (https://github.com/fjruizruano/ngsprotocols/blob/master/repeat_masker_run_big.py, accessed on 14/01/2025) to align the isolated satDNAs against each respective genome, thus giving the relative abundance of one particular satDNA. Kimura-2-parameter (K2P) of each satDNA was calculated by using the script calcDivergenceFromAlign.py in RepeatMasker [39]. All satDNAs were numbered according to their decreasing abundance in the genome and named as suggested by Ruiz-Ruano et al. [33]. Thus, *A. infulatus*, *A. nancymaae*, *A. vociferans*, and *A. trivirgatus* satellites were named AinSats, AnaSats, AvoSats, and AtriSats, respectively.

To check for sex-biased satDNAs in *A. infulatus*, we calculated the male-to-female abundance ratio (M/F) for each AinSat sequence using RepeatMasker outputs. SatDNAs with an M/F ratio greater than 1 were considered male-biased, while M/F ratios below 1 were classified as female-biased.

All recovered satDNAs were submitted to a homology search using a custom Python script- rm_homology.py (https://github.com/fjruizruano/satminer/blob/master/rm_homology.py*)* [33] and were further inspected by manual sequence alignments. SatDNAs were classified according to the similarity criteria proposed by Ruiz-Ruano et al. [33]: variants (> 95% identity), families (80–95% identity), and superfamilies (50–80% identity). Finally, a BLASTn search [40] against the NCBI nucleotide database (GenBank) was conducted to probe for potential homology to previously reported satellite repeats in other species.

### Primer design and polymerase chain reaction (PCR)

Primers were designed for seven AinSatDNAs for which a suitable primer design was possible (Table S1). Primers were manually designed for each satDNA using the Multiple primer analyzer (Thermofisher) (https://www.thermofisher.com/br/en/home/brands/thermo-scientific/molecular-biology/molecular-biology-learning-center/molecular-biology-resource-library/thermo-scientific-web-tools/multiple-primer-analyzer.html*)* and OligoCalc (Biotools)(https://www.biosyn.com/gizmo/tools/oligo/oligonucleotide%20properties%20calculator.htm*)* to avoid hairpin formation and self-annealing. Two AinSatDNAs (AinSat4-34 and AinSat06-34) were synthesized and directly labelled with Cy3 at the 5’ end by ThermoFisher (ThermoFisher Scientific), due to their small repeat unit lengths (RUL) (< 30 bp). PCR reactions were performed in a T100 Bio-Rad Thermal Cycler (Bio-Rad Laboratories) following the conditions described in Kretschmer et al. [21] with some modifications. All reactions consisted of an initial denaturation at 95 °C for 7 min, followed by 34 cycles with denaturation at 95 °C (45 s), annealing temperatures varying from 52 °C to 60 °C (60 s), extension at 72 °C (60 s), and the final extension at 72 °C (7 min).

### Fluorescence in situ hybridization (FISH) and comparative genome hybridization (CGH)

Seven AinSats were fluorescently labeled using a nick-translation kit (Jena Bioscience, Jena, Germany) with Atto550-dUTP and Atto488-dUTP fluorophores, following the manufacturer’s manual. These probes, along with the Cy3-labeled AinSat4-34 and AinSat06-34, were used for Fluorescence in situ hybridization (FISH) on mitotic chromosome spreads from both females and males of *A. infulatus*. In situ hybridizations followed the protocol of Sassi et al. [41] and were conducted under high-stringency conditions. Briefly, we treated the slides with RNAse (10 µg/ml in 2 × SSC solution) for 1 h 30 min at 37 °C and with pepsin (50 µg/mL in 10 mM HCl) for 10 min. Chromosomes were denatured in 70% formamide (72 °C, 3 min, 15 s) and hybridized in a 37 °C moist chamber for at least 14 h.

Additionally, we performed intraspecific CGH using male and female gDNA *of A. infulatus* to check for sex-specific chromosome regions. The gDNAs were labelled using a nick-translation kit (Jena Biosciences) with Atto488-dUTP and Atto550-dUTP. To block repetitive sequences and reduce nonspecific hybridization, we obtained 5 µg of unlabeled C0t-1 DNA from *A. infulatus* and added it to the hybridization mix [42]. We employed the following blocking strategy: female-derived C0t-1 DNA was used for male metaphase spreads, while male-derived C0t-1 DNA was applied to female metaphase spreads. The final mixture contained 500 ng each of labeled male and female gDNA, 5 µg of C0t-1 DNA, and 20 µL of hybridization buffer (50% formamide, 2× SSC, 10% SDS, 10% dextran sulfate, and Denhardt’s buffer, pH 7.0), which was later applied onto male and female mitotic chromosome spreads and hybridized for 72 h. The ratio of probes vs. C0t-1 DNA was chosen based on previous investigations [43–45], and hybridization conditions followed the protocol of Symonová et al. [46].

### Identification of sex chromosomes of *A. infulatus*

The identification of the sex chromosomes in *A. infulatus* was based on a combination of C-banding [47] and Whole Chromosome Painting (WCP) performed subsequently on the slides used for the FISH and CGH assays.

The WCP experiments aimed to highlight the X1 chromosome, using the human X chromosome as a probe. This probe was obtained by microdissection [48] and labeled via DOP-PCR with the Cy-5 fluorophore [48, 49]. These assays followed the protocol of Sassi et al. [41] and employed 5 µg of unlabeled C0t-1 DNA (Invitrogen, California, USA) as a blocking for repetitive sequences. As for the Y chromosome, its identification was based on the presence of three large blocks of constitutive heterochromatin, as previously characterized by Araújo et al. [14]. The precise identification of the X₂ chromosome, however, could not be achieved.

### Microscopy and image processing

We have analyzed a minimum of 30 metaphase spreads per individual to confirm the diploid number (2n), karyotype structure, and FISH results. Slides used for in situ hybridizations were counterstained with 4′,6-diamidino-2-phenylindole (DAPI) solution to confirm chromosome morphology. Images were acquired using an Olympus BX50 microscope (Olympus Corporation, Ishikawa, Japan) equipped with a CoolSNAP camera and processed using Image-Pro Plus 4.1 software (Media Cybernetics, Silver Spring, MD, USA). Chromosomes were classified as metacentric (m), submetacentric (sm), subtelocentric (st), or acrocentric (a) according to the measures of Levan et al. [50].

## Results

### Characterization and comparison of the *Aotus* repeatomes

**Fig. 1:**
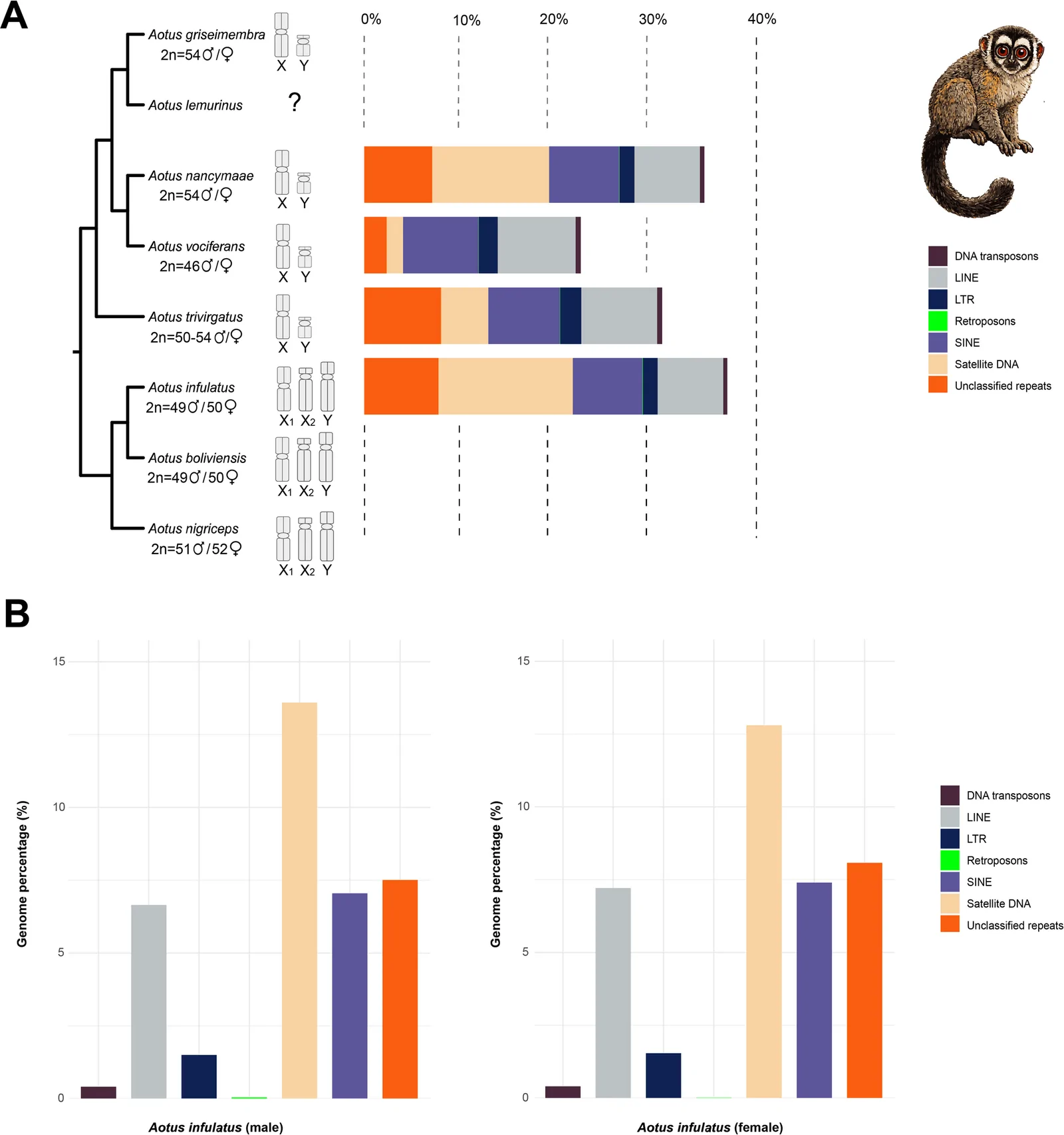
Repeatome composition and phylogenetic context of *Aotus* species. (**A**) Schematic representation of the phylogenetic relationships within *Aotus*, showing the diploid number (2n) of each species, sex chromosome system, and the proportion (%) of repetitive DNA identified in each species analyzed. (**B**) Proportion of repetitive DNA categories between sexes in *Aotus infulatus*. The phylogenetic tree was retrieved from Martins-Junior et al. [17], and cytogenetic data were obtained from Araujo et al. [14] and Ruiz-Herrera et al. [15]. Graphical representations were generated using the R package ggplot2 [37] and combined with the *Aotus* phylogeny using ggtree [38]

Comparative analysis of the repeatomes across *Aotus* species revealed a higher accumulation of repetitive sequences in *A. infulatus* and *A. nancymaae*, which comprised approximately 37% and 34% of their genomes, respectively **(**Fig. 1A; Table 1, Figure S1). In contrast, *A. vociferans* exhibited the lowest proportion (26%), while *A. trivirgatus* showed an intermediate level, with approximately 30% of its genome consisting of repetitive DNA **(**Table 1**)**. Among the different categories of repetitive elements, satDNAs were particularly enriched in *A. infulatus* (13–14%) and *A. nancymaae* (12%), whereas *A. vociferans* and *A. trivirgatus* showed proportions below 5% **(**Fig. 1A; Table 1, Figure S1). Other repetitive elements, including DNA transposons and Retrotransposons, such as Retroposons (i.e., non-LTR retrotransposons that lack the gene encoding reverse transcriptase) LINEs, SINEs, and LTRs, displayed more comparable distributions and divergence patterns across the species analyzed **(**Fig. 1A; Table 1, Figures S1 and S2).

**Table 1:** Proportion of major categories of repetitive DNAs in the genomes of different *Aotus* species

Species	Sex	Repetitive DNA Class (genome %)							Non-repetitive DNA (%)
		DNA transposon	LINE	LTR	Retroposon	SINE	DNA satellite	Unclassified repeats	
*Aotus infulatus*	Male	0.41	6.5	1.47	0.04	7.11	13.98	7.51	63.09
*Aotus infulatus*	Female	0.4	7.21	1.54	0.01	7.4	12.8	8.08	62.48
*Aotus nancymaae*	Male	0.45	6.63	1.52	0.02	7.1	11.84	6.88	65.59
*Aotus vociferans*	Male	0.52	7.88	1.93	0.02	7.64	1.66	2.27	74.12
*Aotus trivirgatus*	Female	0.51	7.69	2.14	0.04	7.23	4.78	7.78	69.74

Comparison of male and female repeatomes in *A. infulatus* revealed an overall similar repetitive DNA landscape between the sexes **(**Fig. 1b; Table 1; Figure S3). However, differences were detected in the abundance of specific categories: LINEs, LTRs, and SINEs were relatively more abundant in females, whereas satDNAs were enriched in the male genome (Fig. 1B; Table 1).

### General features of *Aotus* satellitomes

Iterative analyses with TAREAN revealed an *Aotus* satellitome dominated by nine satDNA families, except for *A. nancymaae* (Tables S2 and S3). Repeat unit lengths (RUL) ranged from 34 bp to 2,956 bp, the latter corresponding to a single satDNA family identified in *A. nancymaae* (AnaSat8-2956). Approximately 63% of the satDNA families possessed monomers exceeding 100 bp (*A. infulatus*, *n* = 6; *A. nancymaae*, *n* = 6; *A. vociferans*, *n* = 5; *A. trivirgatus*, *n* = 5), whereas the remaining families comprised shorter repeats of 34–51 bp. The A + T content varied markedly among satDNA families, ranging from 26.4% to 77%, with only six families exhibiting A + T proportions below 50% (AinSat09-51, AnaSat7-51, AvoSat6-34, AvoSat8-51, AtrSat6-34, and AtrSat8-51). Mean Kimura two-parameter (K2P) divergence values showed moderate interspecific variation, spanning from 7.2 in *A. nancymaae* to 10.9 in both *A. vociferans* and *A. trivirgatus* (*A. infulatus* = 9.7) (Tables S2 and 3, Figures S1-S3). Notably, all satellitomes contained satDNA families with intermediate divergence levels (K2P > 10), including five AinSats, two AnaSats, four AvoSats and five AtrSats.

Comparison of male and female *A. infulatus* satellitomes revealed male-biased abundance for five AinSat families. Three families (AinSat5-288, AinSat6-34, and AinSat8-342) were enriched in males, whereas two (AinSat7-289 and AinSat9-51) showed higher abundance in females (Table S2). Comparative analyses across *Aotus* satellitomes revealed extensive sequence conservation, with > 95% similarity among most satDNA families (Tables S2 and S3, Table 2). Of the 36 satDNA families identified, 12 were shared by all species analyzed, while the remaining families showed overlapping distributions among species subsets (Tables S2 and S3). Four homology groups were identified, indicating shared evolutionary origins of distinct satDNA families. One group (group-2) formed a superfamily (50–80% similarity) comprising AinSat2/AnaSat2/AvoSat2/AtrSat1 and AtrSat1, as well as AinSat3 AnaSat1/AvoSat1/AtrSat2 (Table 2)**.**

**Table 2:** Similarity among AinSatDNA families

Aotus infulatus	Aotus nancymaae	Aotus vociferans	Aotus trivirgatus	Similarity (%)	Homology group
AinSat1-187	AnaSat4-187	AvoSat3-187	-	95–97	Group-1
-	-	-	AtrSat3-145	-	Group-1
AinSat2-185	AnaSat2-185	AvoSat2-185	AtrSat1-185	100	Group-2
AinSat3-344	AnaSat1-344	AvoSat1-344	AtrSat2-344	100	Group-2
AinSat4-34	-	AvoSat5-34	AtrSat5-34	100	Group-3
AinSat5-288	AnaSat3-288	-	AtrSat4-288	98–99	Group-4
AinSat6-34	AnaSat6-34	AvoSat7-34	AtrSat7-34	97–100	Group-3
AinSat7-289	AnaSat5-289	AvoSat4-288	-	100	Group-4
AinSat8-342	-	-	-	-	Group-2
AinSat9-51	AnaSat7-51	AvoSat8-51	AtrSat8-51	100	Group-5
-	-	AvoSat6-34	AtrSat6-34	100	Group-6
-	-	AvoSat9-382	AtrSat9-382	97	Group-4

satDNAs aligned in the same row represent the same family

BLAST analysis showed that this superfamily exhibits high similarity to primate alpha satDNA, including sequences described in *A. azarae*, *Sapajus apella*,* Sapajus cay*,* Callithrix jacchus*,* Saimiri sciureus* and *Saguinus oedipus* (Table S4). Additionally, group-1 (AinSat1-187, AnaSat4-187, AvoSat3-187) is composed of satDNA homologous to heterochromatin repeat OwlRep13 (*A. azarae*).

### Comparative genomic hybridization (CGH) and identification of sex chromosomes

**Fig. 2:**
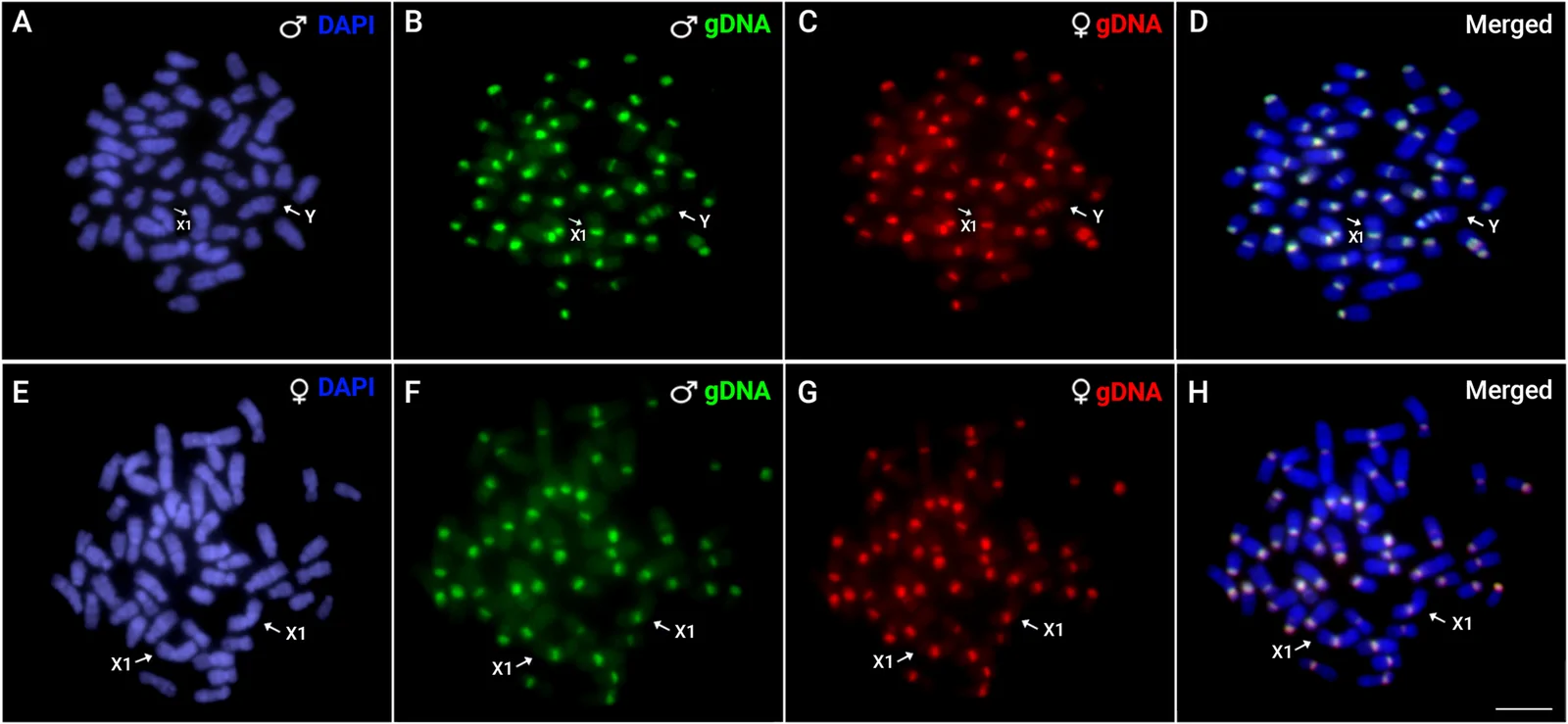
Comparative genomic hybridization (CGH) on metaphase chromosomes of *Aotus infulatus*. CGH on metaphase chromosomes from male (**A**–**D**) and female (**E**–**H**) *Aotus infulatus*. First column (**A**, **E**): metaphases stained with DAPI. Second column (**B**, **F**): hybridization patterns obtained using male genomic DNA (Atto 488, green). Third column (**C**, **G**): hybridization patterns obtained using female genomic DNA (Atto 550, red). Fourth column (**D**, **H**): merged images of genomic DNA probes and DAPI-stained metaphases. Arrows indicate the X₁ and Y sex chromosomes of *A. infulatus.* Scale bar = 5 μm

Intraspecific comparative genomic hybridization (CGH) experiments performed using male and female genomic DNA from *A. infulatus* predominantly revealed hybridization signals in pericentromeric and centromeric regions of all chromosomes, with additional signals detected in interstitial regions in some cases (Fig. 2)**.** Overall, CGH analyses indicated limited genomic differentiation between males and females in *A. infulatus*, with CGH-positive regions shared across the genome. This pattern extended to the sex chromosomes (X_1_ and Y), which showed no sex-specific CGH signals **(**Fig. 2**).** The Y chromosome, however, displayed three conspicuous hybridization bands corresponding to repetitive DNA regions (Fig. 2).

The sex chromosomes in all mitotic metaphases analyzed were identified by C-banding and whole-chromosome painting (WCP) using a human X-chromosome probe, performed sequentially on the same slides following the FISH and CGH experiments (Figures S4 and S5).

### In situ mapping of AinSatDNAs

**Fig. 3:**
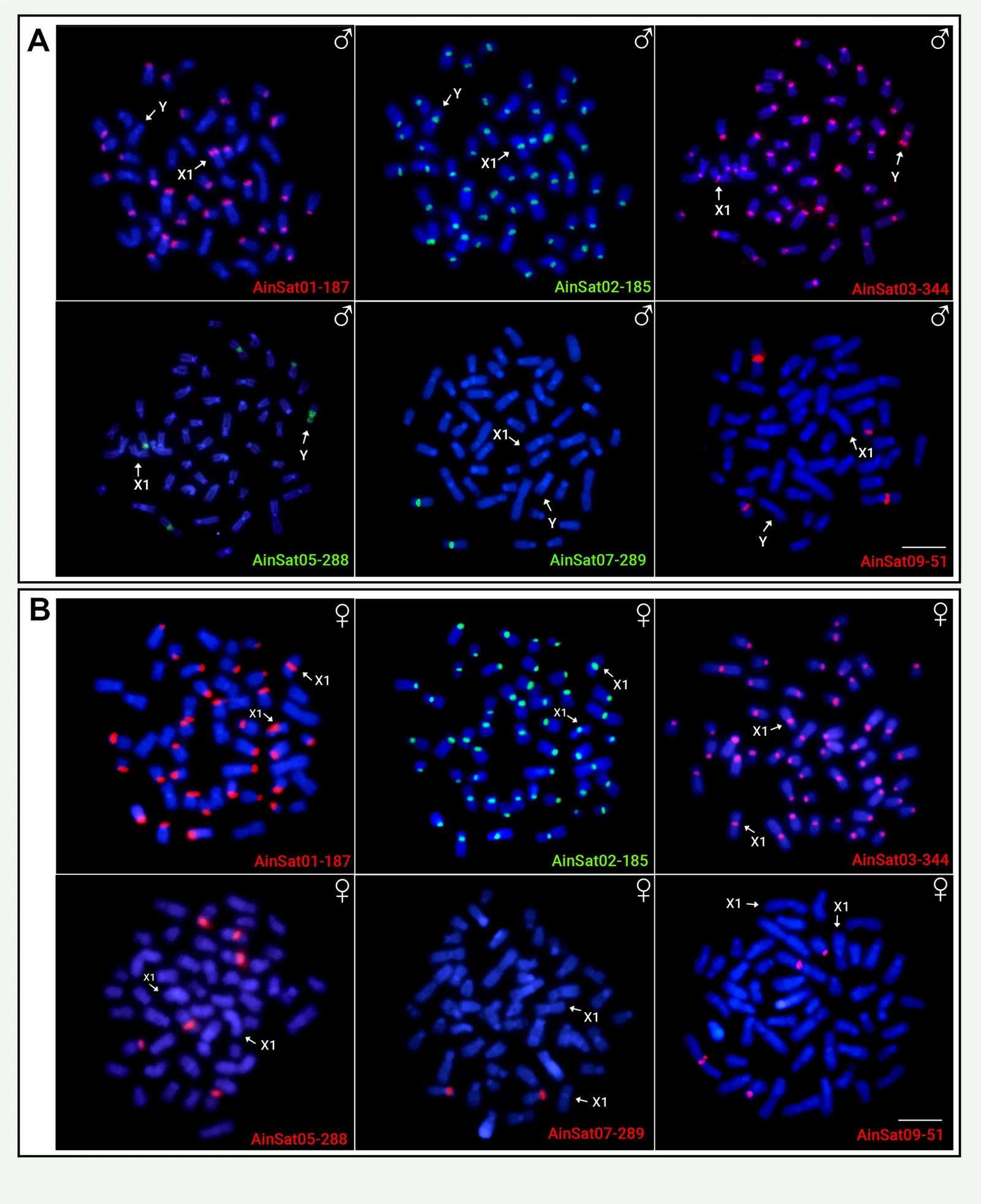
Chromosomal localization of AinSat satellite DNAs in *Aotus infulatus*. FISH on male (**A**) and female (**B**) *Aotus infulatus* metaphases (2n = 49) using AinSat probes. The name of each satDNA is listed in the lower right, corresponding to the fluorochromes used: Atto-488-dUTP (green) and Atto-550-dUTP (red). Arrows indicate the X₁ and Y sex chromosomes of *A. infulatus*. Scale bar = 5 μm

FISH experiments enabled the chromosomal localization of AinSatDNA families, allowing for the assessment of their potential accumulation on the sex chromosomes of *A. infulatus* (Fig. 3).

Of the nine satDNA families identified, six AinSats produced detectable FISH signals **(**Fig. 3**)**. Four AinSatDNAs presented equal distribution of signals between males and females. AinSat02-185 showed centromeric/pericentromeric hybridization signals in all chromosomes, including the X_1_, X_2_, and Y sex chromosomes. AinSat03-344 presented the same patterns observed by AinSat02-185, except for a conspicuous terminal and interstitial cluster displayed on the Y chromosome (Fig. 3). AinSat07-289 and AinSat09-51 had signals in one and two autosome pairs, respectively. The remaining two AinSats displayed sex-specific hybridization patterns. AinSat01-187 showed interstitial/pericentromeric signals on 15 pairs and the X_1_ chromosome in males, with no detectable signal on the Y chromosome, whereas in females, signals were observed on 16 autosome pairs, including both X_1_ chromosomes **(**Fig. 3**).** AinSat05-288 exhibited centromeric/pericentromeric signals on two autosomal pairs and the Y chromosome in males, while in females, signals were detected on three autosomal pairs **(**Fig. 3**).** While we cannot entirely exclude the possibility of interindividual polymorphism, although we have consistently observed this pattern in all males and all females here analyzed, the available data do not allow us to determine the underlying basis of this difference, and multiple biological explanations remain possible. No FISH signals were detected for AinSat4-34, AinSat6-34, or AinSat8-342.

## Discussion

### Repeatome diversification in *Aotus*

Through comparative analyses, we established a representative repeatome profile for four *Aotus* species (*A. infulatus*, *A. nancymaae*, *A. vociferans*, and *A. trivirgatus*). The proportion of genomic sequences assigned to repetitive DNA ranged from 26% to 37% (Fig. 1), falling within the range reported for mammals (approximately 25–50%) [51], (Table 1). Repetitive DNAs are highly dynamic genomic components that exhibit rapid molecular evolution and substantial variation among species [26, 36, 52]. Multiple molecular mechanisms, including segmental duplications, gene conversion, and unequal crossing-over, contribute to the expansion or contraction of specific categories classes of repetitive elements, shaping genome architecture over relatively short evolutionary timescales [26, 53, 54].

Among the major categories of repetitive elements, satDNAs showed the highest degree of interspecific variation, accounting for approximately 5–14% of the genome across *Aotus* species (Fig. 1; Table 1). Despite this quantitative variability, the satellitomes are characterized by a largely conserved repertoire of satDNA families with substantial sequence homology among species. However, marked differences in relative abundance and Kimura-2 divergence values point to rapid sequence evolution and reorganization of these repeats (Tables S2 and S3, Figures S2 and S3). Such dynamics are consistent with the model of concerted evolution, in which satDNAs undergo cycles of lineage-specific amplification and homogenization, resulting in pronounced quantitative divergence even among closely related taxa. Comparable patterns have been documented across a wide range of vertebrates, where satDNA evolution is tightly linked to chromosomal reorganization, centromere evolution, and speciation processes [53, 55–57]. Increasing evidence further suggests that shifts in satDNA composition can influence higher-order chromatin organization and genome stability, thereby contributing to lineage-specific genome architecture and evolutionary trajectories [25, 33, 58].

This evolutionary pattern is consistent with the library hypothesis, which posits that closely related species inherit a common ancestral reservoir of satDNA sequences, with interspecific variation arising primarily through differential amplification, contraction, or epigenetic silencing of individual families [26]. Within this framework, divergence among satellitomes is shaped by both the time elapsed since lineage separation and the degree of historical or ongoing gene flow among populations [26, 59]. In *Aotus*, biogeographic history and divergence timing appear to be particularly influential: the species examined here occupy largely non-overlapping geographic ranges and diverged relatively recently, approximately 4 million years ago [17]. This evolutionary context likely promoted rapid quantitative remodeling of pre-existing satDNA families rather than the de novo emergence and fixation of novel satellite repeats. Accordingly, the repeatome differences observed among *Aotus* species most plausibly reflect the recent species divergence time and the accelerated reorganization of a shared ancestral satDNA repertoire following lineage isolation.

Similar dynamics have been reported in other mammalian and vertebrate groups, where recent divergence combined with geographic isolation facilitates independent copy-number turnover of conserved satDNA families [25, 60–62]. Moreover, stochastic processes such as unequal crossing-over and replication slippage, together with chromosomal rearrangements, can further amplify lineage-specific satellitome divergence over short evolutionary timescales [63, 64]. Emerging evidence also suggests that such rapid satDNA reshaping may have functional consequences for chromatin organization and centromere performance, potentially contributing to postzygotic isolation and speciation [65, 66].

### Evolution of centromeric satDNAs in *Aotus*

SatDNAs are central to genome integrity, particularly through their roles in centromere organization and correct chromosome segregation during cell division [67, 68]. Beyond their structural function, centromeric satellites exhibit rapid sequence turnover and epigenetic plasticity, highlighting the dynamic nature of centromere identity despite conserved chromosomal roles [65, 69].

In this study, we identified eight satDNA families belonging to homology group 2 that likely represent α-satellite variants, characterized by monomer lengths of 185 bp and 344 bp (Table 2). These sequences were identified by BLAST analysis (Table S4), and their centromeric localization was confirmed by FISH in *A. infulatus* (Fig. 3). In primates, α-satellite DNA constitutes the core centromeric repeat and is needed for the recruitment of centromere-specific proteins, including CENP-A and CENP-B, which are required for kinetochore assembly and spindle attachment [52, 68]. Previous studies have shown that alpha satellite architecture differs markedly between New World (Platyrrhini) and Old-World monkeys (Catarrhini): Platyrrhini typically harbor monomers of ~ 340 bp, whereas Catarrhini predominantly possess shorter monomers of ~ 170 bp [20, 52]. Our findings expand this paradigm by demonstrating the coexistence of α-satellite arrays with distinct monomer sizes (185 bp and 344 bp) within four *Aotus* species. BLAST analysis demonstrated that the 344 bp monomer is found in other neotropical monkeys, while the 185 bp unit is mostly found in *Aotus* (Table S4). This observation indicates that the 185 bp might have originated in *Aotus* lineage from the characteristic ~ 340 bp monomer of Platyrrhini, supporting their close evolutionary relationship [19] (Table 2). Comparable evolutionary processes have been documented in other taxa, including alligatorids and some Anostomidae and Symbranchidae fishes, where satDNA monomers are composed of smaller subunits that may persist alongside derived variants [70–72]. In *Aotus*, the simultaneous presence of both monomer sizes suggests that this process may not have been fully fixed, possibly representing a polymorphic state within the lineage [19, 20]. Alternatively, the high sequence identity and coexistence of both monomers may reflect the action of gene conversion and concerted evolution, which are known to homogenize satellite arrays and maintain sequence conservation across genomic regions [26, 52, 73]. Indeed, variation in satDNA composition and abundance has been shown to influence centromere strength and meiotic drive, potentially generating intragenomic conflict and contributing to karyotypic evolution [74, 75]. In mammals, divergence in α-satellite arrays is frequently associated with centromere repositioning and chromosomal rearrangements, reinforcing the link between satDNA evolution and genome architecture [76, 77].

### Sex chromosome–associated satDNA dynamics in *Aotus infulatus*

Comparison of male and female repeatomes in *A. infulatus* revealed an increased proportion of satDNA in the male genome, consistent with a potential role for satDNAs in the differentiation of the X₁X₂Y sex chromosome system. Repetitive DNAs are widely recognized as major drivers of sex chromosome evolution, frequently accumulating on heteromorphic sex chromosomes following the suppression of recombination between homologs [2, 21, 26, 70, 78]. In addition to their role in post-differentiation divergence, satDNAs are known to promote chromosomal rearrangements by providing substrates for non-allelic homologous recombination, facilitating fusions, fissions, and translocations [79–81]. Such repeat-mediated rearrangements have been implicated in the origin of multiple and neo-sex chromosome systems across diverse vertebrate lineages, including primates and other mammals [33, 77, 82]. Together, these observations suggest that satDNA enrichment in the male genome of *A. infulatus* may have contributed not only to sex chromosome differentiation but also to the chromosomal rearrangements underlying the emergence of the X₁X₂Y system.

Among the eight satDNA families isolated, four (AinSat1-187, AinSat2-185, AinSat3-344, and AinSat5-288) showed accumulation on the sex chromosomes of *A. infulatus* (Fig. 3), in agreement with CGH results and heterochromatin distribution patterns (Fig. 2, Figures S4 and S5). Comparative analysis of their chromosomal localization on X1 and Y chromosomes revealed two distinct evolutionary trajectories. The first pattern, exemplified by AinSat1-187 and AinSat2-185, reflects the ancestral configuration of the sex chromosome pair. AinSat2-185 retained its ancestral distribution on both X1 and Y chromosomes, whereas the absence of AinSat1-187 from the Y chromosome likely resulted from post-recombination suppression degeneration, with the sequence being lost from Y but retained on X. This interpretation is supported by FISH data (Fig. 3), AinSat02-185 occupies the same chromosomal position on X1 and Y, whereas the AinSat01-187 showed a shifted FISH signal compared to its position on the X1 chromosome, with no signal detected on the Y chromosome. Notably, AinSat02 is pericentromeric, while AinSat01-187 exhibits terminal signals, a positional difference that could have contributed to the early loss of AinSat01-187 during chromosome degeneration.

The second pattern, represented by AinSat3-344 and AinSat5-288, reflects more recent and dynamic chromosomal events. AinSat3-344 appears to have undergone a chromosomal inversion followed by local amplification (Fig. 3), whereas AinSat5-288 experienced extensive amplification, likely after the formation of the neo-Y chromosome. Together, these patterns indicate that satDNAs have actively contributed to the structural and evolutionary reshaping of the multiple sex chromosome system of *A. infulatus*, acting as dynamic genomic elements that facilitate chromosomal rearrangements and differentiation [24, 57, 78].

## Conclusion

This study provides a comprehensive cytogenomic view of the repeatome associated with the X₁X₂Y sex chromosome system of *Aotus infulatus*. Despite pronounced karyotypic diversity across the genus, *Aotus* species retain a largely conserved satDNA repertoire, consistent with the library hypothesis of satDNA evolution. Divergence among species and between sexes is driven primarily by differential amplification and chromosomal redistribution of pre-existing repeats rather than by the emergence of novel satellite families.

In *A. infulatus*, several satDNAs exhibit male-biased abundance and distinct localization on the X_1_ and Y chromosomes, implicating these repeats in the structural remodeling and differentiation of neo-sex chromosomes following autosome–sex chromosome fusion. These findings underscore the pivotal role of repetitive DNA in mediating chromosomal evolution and demonstrate that repeatome reorganization can proceed rapidly without extensive sequence divergence. However, an important limitation of this study is the absence of a highly contiguous, long-read reference genome for *A. infulatus*, which constrains the resolution of repeat organization, sequence context, and chromosomal architecture. The development of such genomic resources will be essential to determine structural genomic organization and pinpoint the precise sequence boundaries of the autosome-sex-chromosome fusions. Expanding this framework to include additional *Aotus* species (particularly those with simple sex chromosome systems and genomic data from both sexes) will be also essential for better understanding the role of satDNAs in simple versus multiple sex chromosome systems. More broadly, our work positions Owl monkeys as an informative primate system for elucidating the genomic mechanisms underlying multiple sex chromosome evolution.

## Supplementary Information

Below is the link to the electronic supplementary material.

Supplementary Material 1

## Data Availability

Raw sequencing reads of Aotus infulatus were deposited in the sequencing read archive (SRA-NCBI) (https://www.ncbi.nlm.nih.gov/bioproject/PRJNA1400295) under accession numbers SRR36746884 (male) and SRR36746885 (female). Catalogs of satDNAs were deposited on the Genbank with accession numbers: PX852210-PX852218 (A. infulatus), PX852219-PX852226 (A. nancymaae), PX852236-PX852244 (A. vociferans) and PX852227-PX852235 (A. trivirgatus). All other relevant data are included in this published article and its supplementary information files.
