# Supplementary material for: The role of satellite DNAs in repeatome organization and sex chromosome evolution in the owl monkey Aotus infulatus and congeneric species
Source: Biol Direct. 2026 Aug 27;21:156. doi: 10.1186/s13062-026-00948-4 (PMC13520323; doi:10.1186/s13062-026-00948-4)
Supplement: Supplementary file 1 [file 13062_2026_948_MOESM1_ESM.docx]

**Table S1**: List of primers used for amplification of selected AinSatDNAs.

| **AinSatsDNAs** | **Forward primer** | **Reverse Primer** |
| --- | --- | --- |
| AinSat1-187nt | TACCTGCAAATTGCGCTCTC | TTATGACCAATTTCTCTTACCC |
| AinSat2-185nt | CGTGTGTGGATTCAGCAGT | GGTTTAACTCTGGAAGTTGAG |
| AinSat3-344nt | CCATTCAGTTAGATAGCTCC | TTGTCAATGTGTGCATTCAA |
| AinSat5-288nt | GCTAGCAGGCATTTTAGGAC | CGGAAGTTAAGGTGTCTGAC |
| AinSat7-289nt | GCCGGAAGTGAAGGTGTCT | TAGCAGACATTTCAGGGTTTC |
| AinSat8-342nt | AGACTGCATCTAAAACTCAGA | TACTGCTATTTCTTATAGGCT |
| AinSat9-51nt | CCAAGCCAAGCCATTGCAC | CTGGCTTTTTTGCTCTTGTCC |

**Table S2**: General characteristics of the nine satellite DNA families identified in *Aotus infulatus* (AinSat). F = Female, M = Male.

| **satDNA** | **RUL**  **(pb)** | **Abundance F** | **Abundance M** | **Abundance M/F** | **Average of M-F Divergence** | **Divergence**  **M/F** | **A+T**  **(%)** |
| --- | --- | --- | --- | --- | --- | --- | --- |
| AinSat 1-187 | 187 | 0.063952669 | 0.064637083 | 1.010701873 | 4.9 | 1.014314928 | 64.1 |
| AinSat 2-185 | 185 | 0.043157214 | 0.043812604 | 1.015186105 | 3 | 1.073684211 | 54.6 |
| AinSat 3-344 | 344 | 0.035150755 | 0.03418886 | 0.972635154 | 5 | 1.045258621 | 55.2 |
| AinSat 4-34 | 34 | 0.011918507 | 0.013016055 | 1.092087704 | 17.2 | 1.005226481 | 73.5 |
| AinSat 5-288 | 288 | 0.003796685 | 0.006092844 | 1.604779717 | 6.9 | 1.007267442 | 52.8 |
| AinSat 6-34 | 34 | 0.00398113 | 0.004672418 | 1.173641149 | 18.1 | 0.99172642 | 76.5 |
| AinSat 7-289 | 289 | 0.00387207 | 0.00254142 | 0.656346784 | 7 | 1.015804598 | 50.1 |
| AinSat 8-342 | 342 | 0.001268475 | 0.001825486 | 1.439118968 | 14 | 0.989082969 | 62.6 |
| AinSat 9-51 | 51 | 0.001516905 | 0.00122624 | 0.808382362 | 11.3 | 1.046070461 | 47.1 |

**Table S3:** General characteristics of the nine satellite DNA families identified in *Aotus infulatus* (AinSat). F = Female, M = Male.

| **satDNA** | **RUL (bp)** | **Abundance** | **A+T (%)** | **Divergence** |
| --- | --- | --- | --- | --- |
| AnaSat1-344 | 344 | 0.051689309 | 55 | 3.37 |
| AnaSat2-185 | 185 | 0.015371692 | 55 | 2.34 |
| AnaSat3-288 | 288 | 0.009125782 | 53.4 | 6.52 |
| AnaSat4-187 | 187 | 0.004457535 | 64.1 | 5.03 |
| AnaSat5-289 | 289 | 0.002271621 | 50.1 | 6.40 |
| AnaSat6-34 | 34 | 0.001241769 | 74 | 16.30 |
| AnaSat7-51 | 51 | 0.00071448 | 47 | 12.59 |
| AnaSat8-2956 | 2956 | 0.000413694 | 63.1 | 5.54 |
| AvoSat1-344 | 344 | 0.007127450 | 56 | 4.78 |
| AvoSat2-185 | 185 | 0.00597157 | 55 | 2.73 |
| AvoSat3-187 | 187 | 0.004192396 | 64.2 | 4.54 |
| AvoSat4-288 | 288 | 0.0024679 | 54 | 7.26 |
| AvoSat5-34 | 34 | 0.001666273 | 74 | 17.02 |
| AvoSat6-34 | 34 | 0.00114122 | 26.4 | 13.73 |
| AvoSat7-34 | 34 | 0.000652667 | 77 | 17.98 |
| AvoSat8-51 | 51 | 0.000614965 | 47.1 | 15.62 |
| AvoSat9-382 | 382 | 0.000210292 | 59 | 9.84 |
| AtrSat1-185 | 185 | 0.018793835 | 55 | 2.78 |
| AtrSat2-344 | 344 | 0.018606171 | 55.2 | 4.67 |
| AtrSat3-145 | 145 | 0.012622609 | 65 | 10.56 |
| AtrSat4-288 | 288 | 0.00457705 | 54 | 7.16 |
| AtrSat5-34 | 34 | 0.003739220 | 74 | 17.00 |
| AtrSat6-34 | 34 | 0.001429336 | 27 | 14.32 |
| AtrSat7-34 | 34 | 0.001265076 | 74 | 17.76 |
| AtrSat8-51 | 51 | 0.000610870 | 47.1 | 15.06 |
| AtrSat9-382 | 382 | 0.000299819 | 58.4 | 8.63 |

**Table S4:** BLAST-search results from *Aotus* satellite DNAs against GenBank-NCBI database.

| **SatDNA family** | **BLAST-Sequence name** | **BLAST- Species ID** | **Query-cover (%)** | **E-value** | **Percentage identity (%)** | **Accession number** |
| --- | --- | --- | --- | --- | --- | --- |
| AinSat1-187 |  |  |  |  |  |  |
|  | Heterochromatin repeat pFosOwlRep13 | *Aotus azarae* | 100 | 7e-81 | 97.3 | AB746944.1 |
| AinSat2-185 |  |  |  |  |  |  |
|  | OwlAlp1 | *Aotus azarae* | 100 | 3e-89 | 100 | LC456013.1 |
| AinSat3-344 |  |  |  |  |  |  |
|  | OwlAlp2 | *Aotus azarae* | 100 | 1e-171 | 99.1 | LC456046.1 |
| AinSat8-342 |  |  |  |  |  |  |
|  | Satellite alpha | *Aotus azarae* | 99 | 7e-54 | 78.2 | LC002884.1 |
|  | OwlAlp2 | *Aotus azarae* | 99 | 9e-53 | 78.6 | LC456044.1 |
|  | Satellite alpha | *Sapajus apella* | 96 | 1e-51 | 78.7 | LC075879.1 |
|  | Satellite alpha | *Sapajus cay* | 56 | 6e-35 | 81.9 | OR838661.1 |
| AnaSat1-344 |  |  |  |  |  |  |
|  | OwlAlp2 | *Aotus azarae* | 100 | 8e-173 | 99.4 | AB762007.1 |
|  | Satellite alpha | *Callithrix jacchus* | 90 | 9e-63 | 82 | LC075852.1 |
|  | Satellite alpha | *Saimiri sciureus* | 75 | 7e-54 | 82.6 | LC075931.1 |
|  | Satellite alpha | *Saguinus oedipus* | 81 | 3e-53 | 81.3 | LC075957.1 |
| AnaSat2-185 |  |  |  |  |  |  |
|  | OwlAlp1 | *Aotus azarae* | 100 | 3e-89 | 100 | LC455998.1 |
|  | Satellite alpha | *Saguinus oedipus* | 45 | 8e-16 | 88 | LC075959.1 |
| AnaSat4-187 |  |  |  |  |  |  |
|  | Heterochromatin repeat pFosOwlRep13 | *Aotus azarae* | 100 | 7e-81 | 97.3 | AB746944.1 |
| AvoSat1-344 |  |  |  |  |  |  |
|  | OwlAlp2 | *Aotus azarae* | 100 | 2e-173 | 99.4 | AB762007.1 |
|  | Satellite alpha | *Callithrix jacchus* | 96 | 7e-64 | 81 | LC075852.1 |
|  | Satellite alpha | *Saguinus oedipus* | 86 | 3e-58 | 81.4 | LC075957.1 |
|  | Satellite alpha | *Saimiri sciureus* | 81 | 1e-56 | 81.9 | LC075931.1 |
| AvoSat2-185 |  |  |  |  |  |  |
|  | OwlAlp1 | *Aotus azarae* | 100 | 3e-89 | 100 | LC456013.1 |
|  | Satellite alpha | *Saguinus oedipus* | 70 | 3e-20 | 82.4 | LC075959.1 |
| AvoSat3-187 |  |  |  |  |  |  |
|  | Heterochromatin repeat pFosOwlRep13 | *Aotus azarae* | 100 | 1e-83 | 98 | AB746944.1 |
| AtrSat1-185 |  |  |  |  |  |  |
|  | OwlAlp1 | *Aotus azarae* | 100 | 5e-175 | 97.3 | LC456009.1 |
|  | Satellite alpha | *Saguinus oedipus* | 84 | 6e-25 | 80.4 | LC075959.1 |
| AtrSat2-344 |  |  |  |  |  |  |
|  | OwlAlp2 | *Aotus azarae* | 100 | 3e-89 | 97 | LC456046.1 |
|  | Satellite alpha | *Callithrix jacchus* | 98 | 1e-123 | 80.3 | LC075852.1 |
|  | Satellite alpha | *Saguinus oedipus* | 84 | 2e-106 | 79.4 | LC075957.1 |
|  | Satellite alpha | *Saimiri sciureus* | 88 | 5e-103 | 78.5 | LC075931.1 |
| AtrSat3-145 |  |  |  |  |  |  |
|  | Heterochromatin repeat pFosOwlRep13 | *Aotus azarae* | 100 | 5e-75 | 91.2 | AB746944.1 |

**Figure S1**: Proportion of repetitive DNA classes between three *Aotus* species: *A. nancymaae* (A), *A. vociferans* (B), *A. trivirgatus* (C).

**Figure S2:** Repeat landscapes showing the divergence values of satellite DNAs and transposable elements in three *Aotus* species: *A. nancymaae* (A), *A. vociferans* (B), *A. trivirgatus* (C).

**Figure S3**: Repeat landscapes showing the divergence values of satellite DNAs and transposable elements in males (A) and females (B) of *Aotus infulatus*.

**Figure S4:** Whole chromosome painting (WCP), using the X chromosome probe (Cy5), and C-banding experiments in mitotic chromosomes of males of *Aotus infulatus*, previously used in the FISH experiments of Figures 2 and 4. Arrows indicate the location of the X_1_ and Y chromosomes. Bar= 5μm

**Figure S5:** Whole chromosome painting (WCP), using the X chromosome probe (Cy5), and C-banding experiments in mitotic chromosomes of females of *Aotus infulatus*, previously used in the FISH experiments of Figures 3 and 4. Arrows indicate the location of the X_1_ chromosome. Bar= 5μm
